# Gold nanoparticle–cellulose/PDMS nanocomposite: a flexible dielectric material for harvesting mechanical energy†

**DOI:** 10.1039/c9ra10811d

**Published:** 2020-03-10

**Authors:** Manojit Pusty, Parasharam M. Shirage

**Affiliations:** Discipline of Metallurgy Engineering and Materials Science, Indian Institute of Technology Indore Simrol Indore 453552 India paras.shirage@gmail.com pmshirage@iiti.ac.in +91-787-5222-331

## Abstract

Cellulose is an abundant natural piezoelectric polymer and is also a renewable resource of significant importance. Here in this work we realize an enhanced piezoelectric response with cellulose in a polydimethylsiloxane (PDMS) matrix by forming a nanocomposite with the incorporation of gold nanoparticles (Au NPs). In the Au NP–cellulose/PDMS nanocomposite an enhancement in the dielectric constant is recorded due to the presence of cellulose alone and a reduction of dielectric loss is found owing to the presence of Au NPs. This opens the possibility of realizing a nanodielectric material from the nanocomposite under current study. This also indicates the significant potential of the nanocomposite towards energy conversion applications. Subsequently, a mechanical energy harvesting device was fabricated using the Au NP–cellulose/PDMS nanocomposite, which is named as a piezoelectric nanogenerator (PNG). The PNG delivered an enhanced open circuit voltage of ∼6 V, short circuit current of ∼700 nA and a peak power density of 8.34 mW m^−2^ without performing any electrical poling steps. The PNG could charge a 10 μF capacitor to 6.3 V in 677 s and could light two commercial blue light emitting diodes (LEDs) simultaneously. The PNG exhibited a good energy conversion efficiency of 1.8%. A touch sensor application of the PNG is also shown.

## Abstract 2

A gold nanoparticle–cellulose/PDMS based piezoelectric nanogenerator (PNG) is reported in this work. The PNG delivers an efficiency of 1.8%. It can light LEDs and sense finger touch.

## Graphical abstract

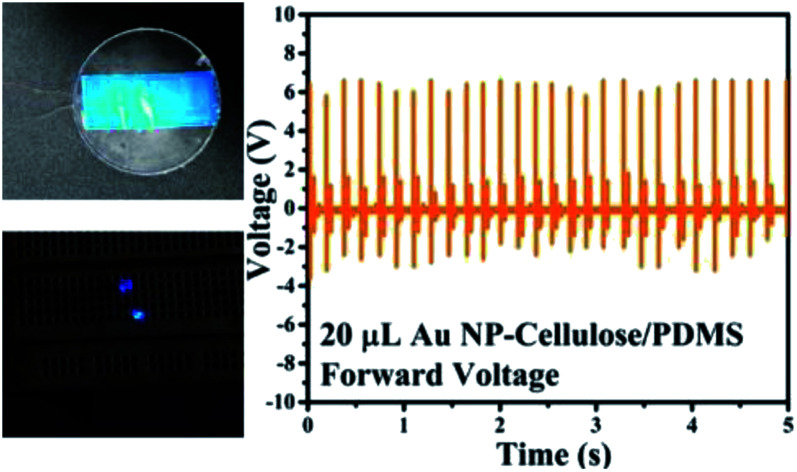

## Introduction

Mechanical energy harvesting is a popular research trend these days after Z. L. Wang showed for the first time the generation of electrical energy from ZnO nanowires (ZnO NWs) and coined the term nanogenerator, which opened the pathway for the development of energy harvesters following the green path.^1^ This was followed by a lot of work done on piezoelectricity assisted mechanical energy harvesting using nanomaterials.^2–9^ Flexibility is a key issue in mechanical energy harvesting applications as it opens up the way for a broad range of applicability to practically realize a body/apparel attachable energy harvester. Previously it was reported that composite based materials show good mechanical energy harvesting performance.^10–12^ Polydimethylsiloxane (PDMS) is often used as a host polymer to form a piezoelectric composite because of its low Young's modulus, adequate biocompatibility, excellent transparency, cost-effectiveness, and good thermal and chemical stability.^13,14^ Some of the naturally occurring piezoelectric materials are bones, hair, collagen fibrils, peptides, cellulose, sugar cane, *etc*.^15^ Among them cellulose is a piezoelectric material that is obtained abundantly in nature in its natural form and is eco-friendly and biocompatible. Cellulose has low density, high mechanical strength, thermal stability and chemical resistance. Also, cellulose exhibits both direct and converse piezoelectricity.^16^ Cellulose is composed of crystallites along with the presence of amorphous regions of low degree of order.^17,18^ Several works were reported for energy harvesting applications using cellulose. The piezoelectric energy harvesting using porous nano fibrils of cellulose were shown by Zheng *et al.*^19^ It was found from the previously reported works that the synthesis of cellulose nanofibrils (CNF) follows specialized synthesis techniques which are neither simple nor cost effective.^20^ Recently, Alam *et al.* conducted an excellent research work to show the fabrication of native cellulose based piezoelectric generator by the incorporation of carbon nanotubes (CNTs) in PDMS matrix.^14^ Native cellulose exists in two forms cellulose I and cellulose II. Cellulose I is crystalline and has scattered amorphous regions of low degree of order, whereas cellulose II requires further chemical treatments to prepare. In this work native cellulose powder is used that is comprised of microfibrils of cellulose and it is easily available in the market. This approach keeps the synthesis process simple and cost effective. Nanostructures such as ZnS,^21^ SbSI,^22^ BaTiO_3_,^23^ ZnO^24^ were reported to be used with cellulose for piezoelectric energy harvesting purpose, but the output voltage was less compared to our results. The advantages of incorporating nanostructures in polymer are that the energy conversion efficiency significantly enhances. This is because the high surface to volume ratio as well as other surface effects plays a dominant role as compared to the bulk, which leads to a greater deformation of the piezoelectric nanostructures comparatively by smaller forces.^8,25^ Noble metal nanoparticles which are biocompatible and are easy to synthesize were previously added in synthetic piezoelectric polymers like PVDF.^26^ It is well known that incorporation of nanostructured metal oxides in PVDF results in the nucleation and stabilization of polar piezoelectric phases.^27^ It is also known that the dipole polarization relaxation mechanism is enhanced due to the addition of nano-particles in a polymer nanocomposite.^28^ Due to these features gold nanoparticles (Au NPs) are selected as nano-fillers to be incorporated in PDMS along with cellulose to form a nanocomposite. Also, working with Au NPs have several advantages like they are chemically stable, easy to synthesize and shows good reproducibility.

In this work native cellulose powder and Au NPs are added in PDMS to form a nanocomposite. It is found that the dielectric constant of cellulose/PDMS rises manifold compared to pure PDMS. Whilst the dielectric constant of the Au NP–cellulose/PDMS is found to be in the similar range, however it is found that the dielectric loss decreases. Such a material can also be used as a dielectric capacitor. Dielectric capacitors have high power density owing to the very high rate of energy uptake and release. They have very wide applications in hybrid electric vehicles, medical fields, future weapon systems *etc*.^29^ Ceramics based dielectric materials have several disadvantages like small breakdown strength, high density and requires advanced processing conditions. Although the dielectric constant of the Au NP–cellulose/PDMS nanocomposite is not greater than 10, however it has several distinct advantages as compared to ceramic based dielectric materials. The Au NP–cellulose/PDMS nanocomposite not only shows excellent flexibility but is also easy to process and can be molded in any shape or size depending on specific applications. Cellulose is an abundant natural polymer, which is biodegradable due to which it might be specifically suitable for medical applications. Some of the other advantages of incorporating Au NPs as nano-fillers are that they render low filler loading due to which some of the inherent polymer properties like density, flexibility and easy processability are not compromised.

Further, in this work a piezoelectric mechanical energy harvesting device is fabricated using the Au NP–cellulose/PDMS nanocomposite which is named as piezoelectric nanogenerator (PNG). It is found that the incorporation of Au NPs in cellulose/PDMS enhances the open circuit voltage as compared to cellulose/PDMS which is attributed to the interaction of cellulose to the large surface area of the Au NPs. Without any external poling the PNG delivered an open circuit voltage of 6 V, when periodically excited by a force of 3 N generated by a mechanically operated external source. The PNG could charge a 10 μF capacitor in 677 s and showed an energy conversion efficiency of 1.8%. The PNG could light two commercial blue light emitting diodes (LEDs). Also, a touch sensing application of the PNG is shown in this work.

## Experimental section

### Synthesis of Au nanoparticle seeds^30^

To prepare gold nanoparticle seeds, 200 μL of 1 M NaOH (Alfa Aesar, India) was added to 100 mL of 1 mM HAuCl_4_·3H_2_O (Alfa Aesar, India) and the solution was boiled. The solution was stirred with a PTFE coated magnetic stirrer at 300 rpm. After a while, 38.8 mM Na_3_Ctr·2H_2_O (Merck, India) was added to the solution quickly. After some time, the colour of the solution turned wine red. Now deionized (DI) water was added to the solution to make it 100 mL final volume.

### Synthesis of Au nanoparticles

10 mL seed solution from the previous step was added to a clean beaker, within which 227 μL of 44.7 mM HAuCl_4_·3H_2_O was added and the solution was boiled under constant stirring at 300 rpm. After that 176 μL of 38.8 mM Na_3_Ctr·2H_2_O was added. The reaction process continued for half an hour.

### Fabrication of piezoelectric nanogenerator (PNG)

**Fig. 1:**
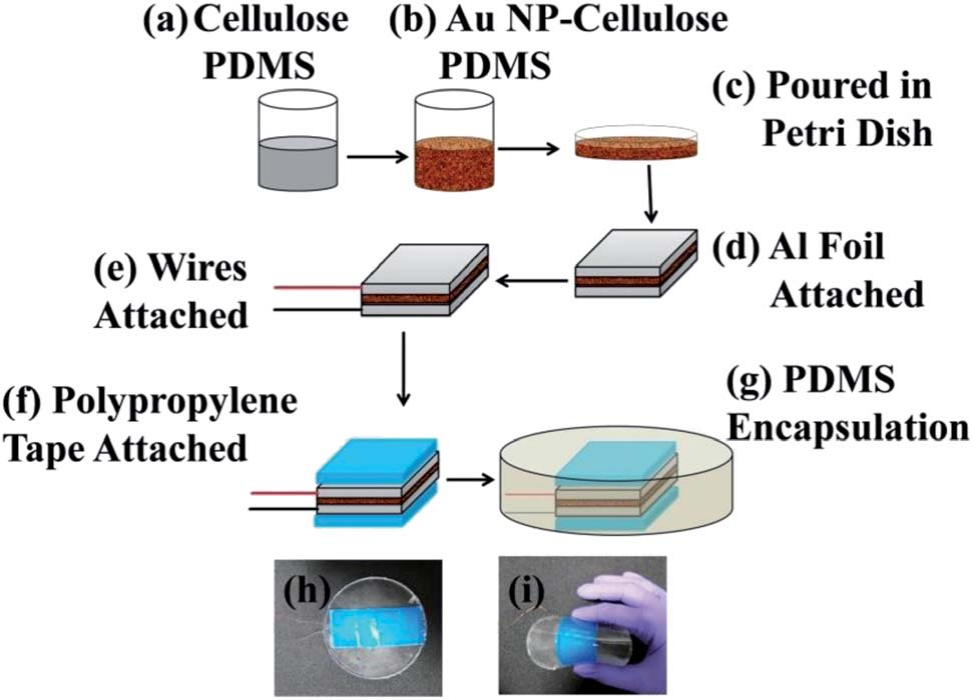
Schematic diagram of the step-wise fabrication process of the PNG. (a) Cellulose and PDMS were mixed in a beaker, (b) within the mixture of cellulose and PDMS varied quantity of Au NP colloid was added, (c) the mixture was poured in a Petri dish and was put in an electric furnace for curing, (d) after curing the Au NP–cellulose/PDMS nanocomposite was cut and aluminium electrode was attached to the top and bottom of the composite, (e) after that polypropylene (PP) tapes were attached on the top to completely cover the aluminium electrodes, (f) conducting wires were attached to both the surfaces, (g) the nanocomposite was encapsulated by PDMS, (h) optical image of the as fabricated PNG, (i) optical image showing the flexibility of the PNG.

10 g of PDMS (10 : 1, PDMS and curing agent) was taken in a beaker, within which 500 mg of cellulose was added (Loba Chemie, India). After that 2, 20, 200 μL colloid of gold nanoparticle was added to the previous mixture. Also, a cellulose and PDMS mixture was prepared, without addition of gold nanoparticles for comparison purpose. The mixture was poured in a Petri dish and was placed in an electric furnace at a temperature of 70 °C, for 1 hour. This step was necessary for the curing/hardening of the PDMS mixture. After the curing process is completed the composites were cut in 3.5 × 8.5 cm dimensions. Aluminium tapes of 3 × 8 cm dimensions with conductive adhesives were attached on top of the as cut composite. Conductive wires were attached to the adhesive of the aluminium tapes. Then the aluminium electrodes and the wires were completely covered with polypropylene tapes. This step was done to eliminate triboelectric effect arising due to direct contact between the aluminium electrodes and the outer PDMS encapsulation. After this stage the device was placed in a Petri dish and was encapsulated by a layer of PDMS to protect the device from mechanical stress, environmental factors like humidity to ultimately increase its longevity. The electrical poling process is ruled out in the fabrication process of the PNG. The complete schematic of the fabrication of the PNG is shown in Fig. 1.

### Characterizations and measurements

The morphological characterization was done by XRD (Bruker D8 Advance X-ray diffractometer) with Cu-K_α_ radiation (*λ* = 1.54 Å). The surface morphology of the cellulose microfibrils and the cellulose/PDMS nanocomposite was carried out by field emission scanning electron microscope (Supra 55 Zeiss). The surface morphology of the gold nanoparticles was carried out by high resolution transmission electron microscopy (JEOL, JEM – 2100). Fourier transform-infra red spectroscopy measurement was carried out by FT-IR Bruker Tensor 27 spectrophotometer. The ultraviolet-visible spectroscopy measurement was carried out by UV-visible Agilent Cary-60 UV-vis spectrophotometer. The dielectric constant *vs.* frequency characteristics is measured by NF ZM2376 LCR Meter. The open circuit voltage signals and the short circuit current signals were measured using a digital storage oscilloscope (Scientific SMO502). The d_33_ measurements were carried out by SIN0CERA YE2730A d_33_ Meter. The PFM measurements were carried out by Park Systems NX10 Atomic Force Microscope. The stress *vs.* strain characteristics were measured by Anton Paar Physica MCR 301 rheometer.

## Results and discussion

**Fig. 2:**
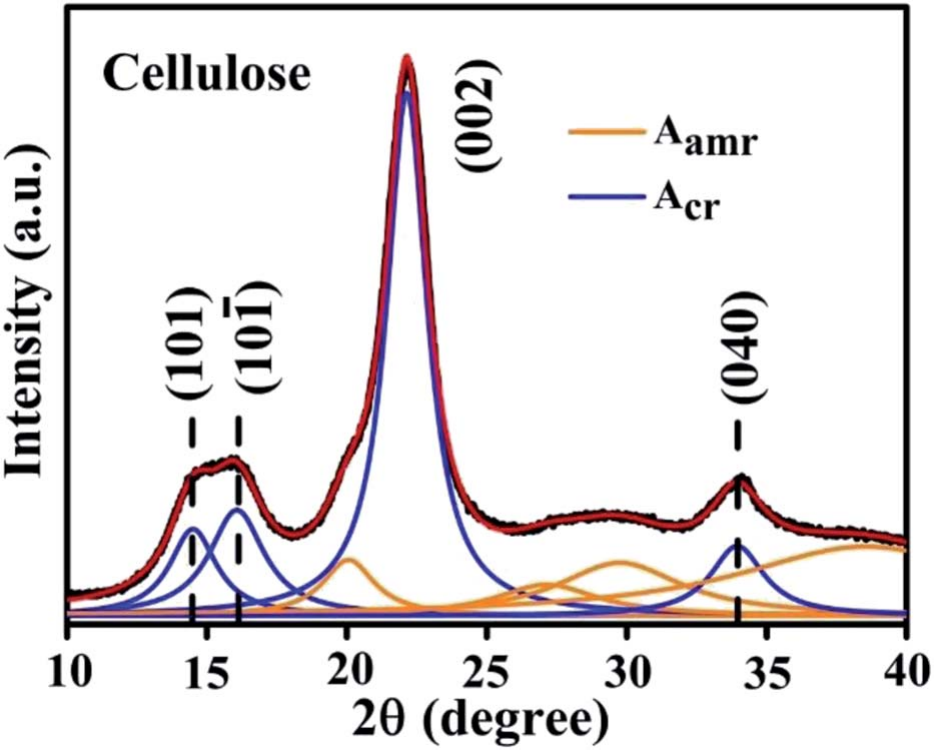
XRD spectra of cellulose powder.

The X-ray diffraction (XRD) spectra of pure cellulose is shown in Fig. 2. The XRD spectra of pure cellulose matches with cellulose I. Cellulose I show peaks at 14.7°, 16.36°, 22.38° and 34.28° corresponding to (101), (101̄), (002) and (040) planes, respectively. The crystallinity shown by pure cellulose is found to be 62.6%. The degree of crystallinity of the cellulose used is calculated from the following equation:^14^1
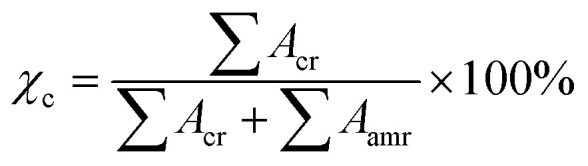
where ∑*A*_cr_ = total integral area of crystalline peaks, ∑*A*_amr_= total integral area of amorphous halos.

**Fig. 3:**
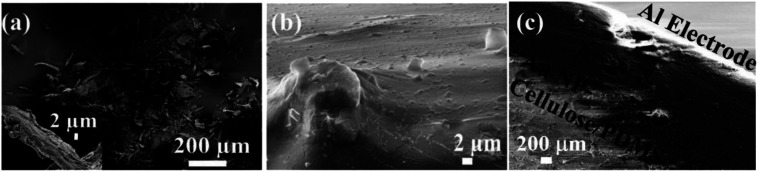
Shows the FE-SEM image of (a) cellulose microfibrils, the inset shows single microfibrils of cellulose, (b) surface of the Au NP–cellulose nanocomposite, indicating the complete encapsulation of cellulose microfibrils by PDMS, (c) cross section of the Au NP–cellulose/PDMS based PNG.

Fig. 3(a) shows the field emission-scanning electron microscopy (FE-SEM) image of the cellulose powder, where the cellulose powder is comprised of cellulose microfibers and some agglomerated microfibers. The figure in the inset shows the magnified image of one microfiber which has a diameter of roughly 5 μm. Fig. 3(b) shows the surface of the Au NP–cellulose nanocomposite, where the cellulose microfibrils are completely encapsulated by the PDMS. This indicates the formation of a homogenous nanocomposite of Au NPs, cellulose and PDMS. Fig. 3(c) shows the FE-SEM image of the cross-sectional area of the Au NP–cellulose/PDMS based PNG device. It can be observed from the cross-sectional FE-SEM image that the aluminium electrode is fixed without any gaps on the Au NP–cellulose/PDMS nanocomposite. The region of the nanocomposite that is near to the aluminium electrode is darker in shade which may be due to greater extent of curing owing to its proximity to the top surface. The roughness on the surface of the cross section of the nanocomposite is due to the presence of cellulose microfibrils. The voids that are visible in the image may be introduced due to the displacement of the rigid cellulose microfibrils that was present at the interface while cutting the nanocomposite with a scissors during FE-SEM sample preparation. This can be confirmed from the fact that before curing the nanocomposite was placed in vacuum for complete removal of trapped air bubbles.

The surface plasmon resonance (SPR) which is a prominent spectroscopic phenomenon that is observed in Au NPs is also found to be linked to the size of the Au NPs. Au NPs with diameter less than 50 nm absorbs light, whereas the Au NPs with larger diameter scatters incident light.^31^ The reason behind this phenomenon is that the size of the NPs are considerably smaller than the wavelength of the incident light due to which the distribution of the light and consequently the charges formed due to the polarization, both are uniform. In Au NPs with diameter greater than 50 nm scattering leads to the damping of the motion of the electrons. The relation between the NP size and the full width at half maximum (FWHM), *Γ* of the SPR band is given by the following equation:^32^2
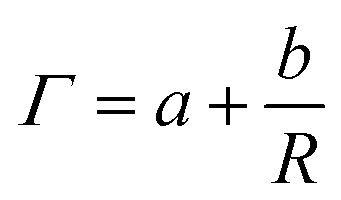
where *a* and *b* are material constants and *R* is the radius of the NP. With the increase in size of the diameter of the NPs, the fraction of electrons close to the surface of the NPs represents a smaller fraction of the total oscillating electrons and hence the total damping is reduced. Thus, the NP size plays an important role in the light dependent polarization of the Au NPs. In this work the size of the Au NPs are less than 50 nm which indicates that the Au NPs are SPR active.

**Fig. 4:**
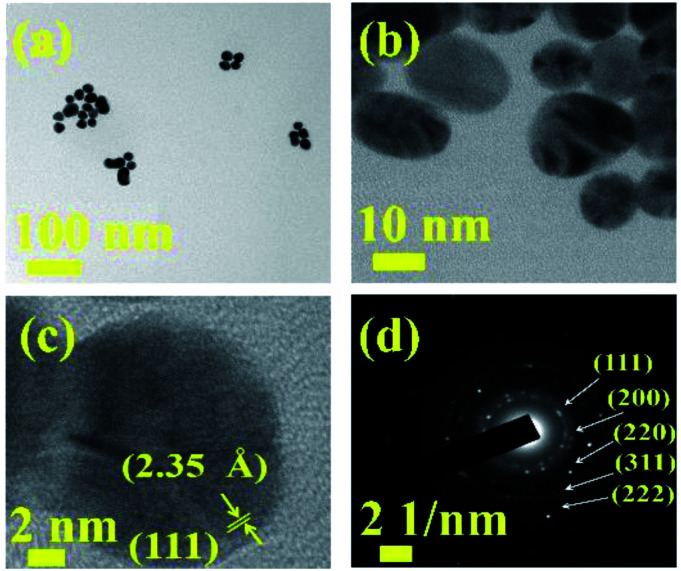
HRTEM images of Au NPs at (a and b) two different magnifications, (c) lattice fringes, (d) SAED pattern corresponding to fcc Au.

The morphological characterization of the synthesized Au NPs were carried out by high resolution-transmission electron microscopy (HR-TEM). The HR-TEM characterization shows aggregated Au NPs in Fig. 4(a and b). The nanoparticles are spherical in shape with an average diameter of ∼15 nm. The lattice fringes of a single Au NP in the HR-TEM image as shown in Fig. 4(c) indicates a *d*-spacing of 2.35 Å corresponding to (111) plane of fcc Au. The SAED pattern as shown in Fig. 4(d) shows polycrystalline behaviour of the sample and the pattern can be indexed to fcc phase of metallic Au.^30^ The Au NPs were synthesized in this work excluding any surfactant.

**Fig. 5:**
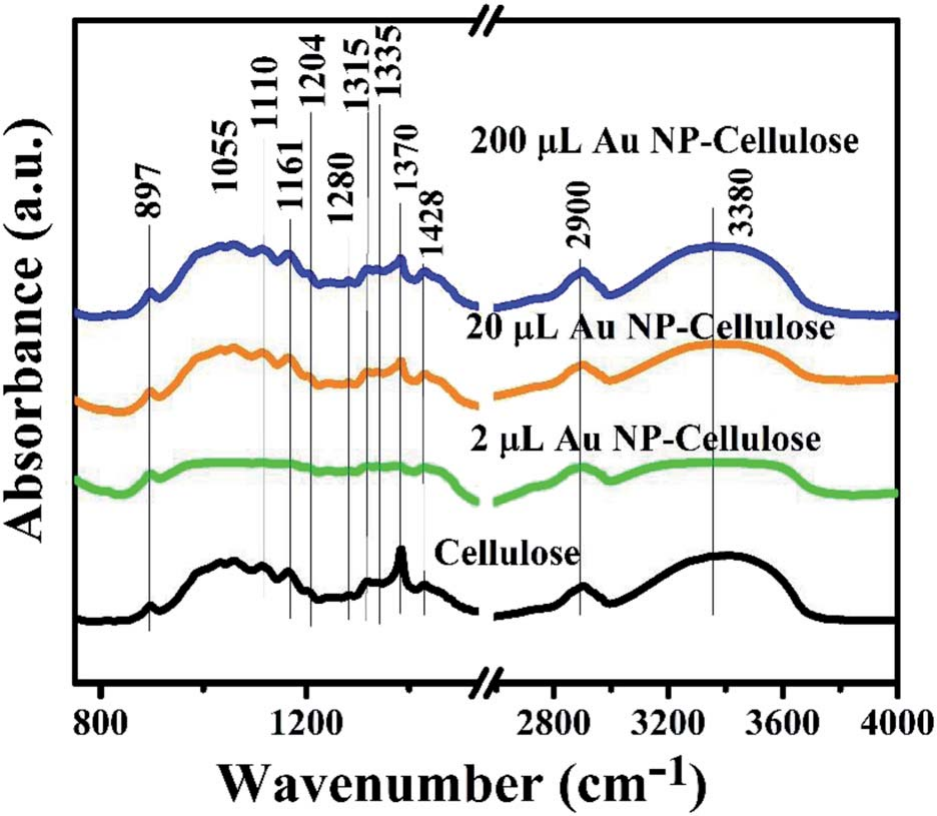
Shows the ATR-IR spectra of pure cellulose powder, 200 μL Au NP–cellulose, 20 μL Au NP–cellulose and 2 μL Au NP–cellulose.

The infra-red (IR) spectroscopy in attenuated total reflection (ATR) mode was performed to look out for possible changes in the cellulose structure by the inclusion of Au NPs. The ATR-IR spectra of pure cellulose, 2 μL Au NP–cellulose, 20 μL Au NP–cellulose and 200 μL Au NP–cellulose are shown in Fig. 5. The ATR-IR spectra indicates a reduction in the peak intensity of 1370 cm^−1^ of cellulose with the addition of 2 μL Au NP. The peak at 1370 cm^−1^ belongs to the COH vibration of cellulose. The peak reduction can be attributed to the formation of hydrogen bonds between the OH functional groups of cellulose and Au NPs, at the expense of the intrinsic COH bonds that were already present in the pure cellulose. However, the same peak is visible with the addition of 20 μL Au NP and 200 μL Au NP, although it is slightly diminished. This may be attributed to the agglomeration of Au NPs in PDMS matrix. The assignment of the vibrational bands of cellulose I is provided in Table 1.

**Table 1:** Assignment of vibrational bands of cellulose I determined by IR spectroscopy in ATR mode

Sl. No.	Wavenumber (cm^−1^)	Band assignment
1	897	COC asymmetric stretching
2	1055	CO stretching vibrations
3	1110	Asymmetric vibration of glucose ring
4	1161	COC asymmetric vibration
5	1204	COH in-plane at C-6 bending
6	1281	CH and OH vibration
7	1315	COH and HCC vibration
8	1335	OH and CH_2_ vibration
9	1370	COH and HCC vibration of cellulose and hemicelluloses
10	1428	CH_2_ vibration; HCH and OCH in plane banding; intermolecular hydrogen bond bending
11	2900	CH and CH_2_ vibration presence in aliphatic methylene groups
12	3340	Stretching vibrations of CH and OH groups

**Fig. 6:**
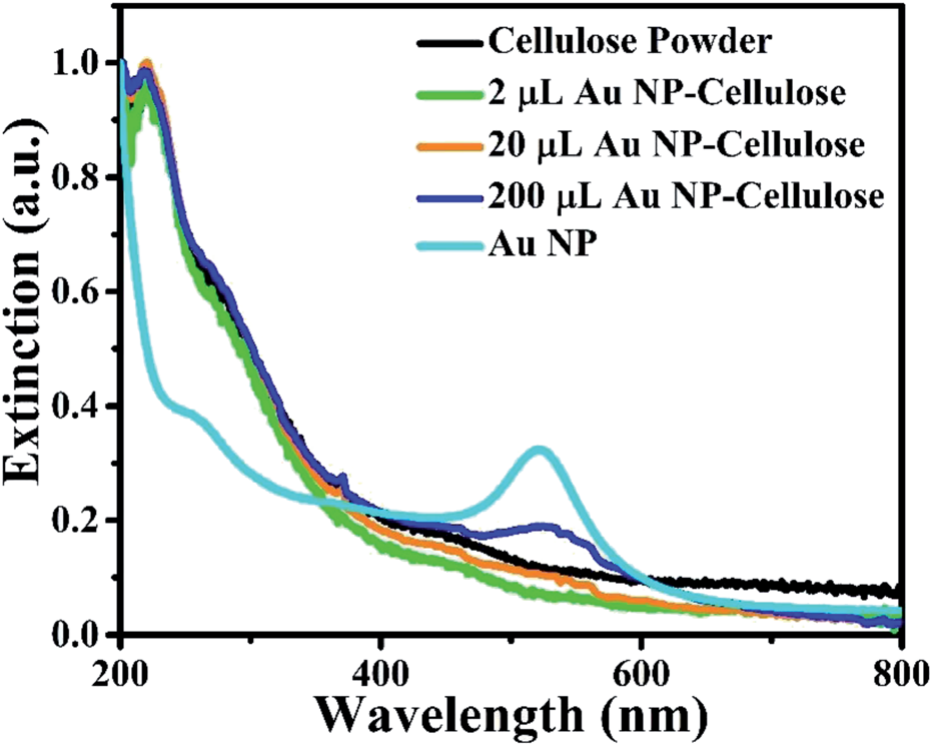
UV-visible spectra of pure cellulose powder, 2 μL Au NP–cellulose, 20 μL Au NP–cellulose, 200 μL Au NP–cellulose and Au NPs.

The ultra violet-visible (UV-vis) spectra of Au colloid, pure cellulose, 2 μL Au NP–cellulose, 20 μL Au NP–cellulose and 200 μL Au NP–cellulose are shown in the Fig. 6. It is observed that there is no absorption in the UV-vis spectra in the visible region for the microcrystalline pure cellulose sample. However, there is a small amount of absorption in the UV spectra at wavelengths less than 400 nm. The UV-vis spectra of pure Au NPs shows an absorption peak near 520 nm, which is attributed to the SPR bands of the Au NPs. The light is not completely absorbed in the process of promoting the electrons from ground state to the excited state. Some light is absorbed in the process of destructive interference between the excited displacement electrons which are in oscillating state and the light itself. For 200 μL Au NP–cellulose sample, the SPR band is detected at the same wavelength of 520 nm. The SPR band is almost diminished in the 20 μL Au NP–cellulose and 2 μL Au NP–cellulose nanocomposite sample. A gradual peak shift towards higher wave numbers can be observed in the Au colloid, 200 μL Au NP–cellulose and 20 μL Au NP–cellulose samples. The shifting of the peaks to the higher wavenumbers indicate agglomeration of Au NPs.^33^ UV-vis study shows an important observation that the 20 μL Au NP–cellulose and 2 μL Au NP–cellulose nanocomposites are SPR inactive. This may be attributed to the fact that the cellulose microfibrils blocks the UV and visible lights to interact with the Au NPs that are present in the nanocomposite.

**Fig. 7:**
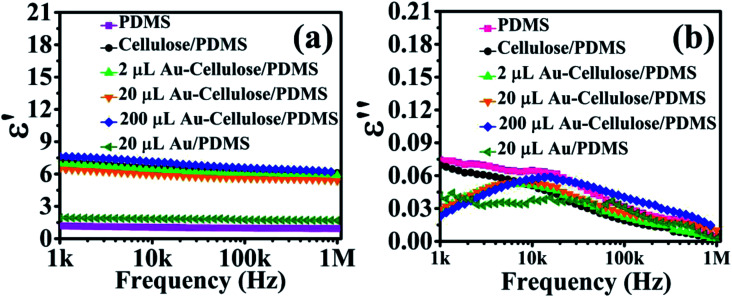
Shows the (a) dielectric constant *vs.* frequency, (b) dielectric loss *vs.* frequency, characteristics of PDMS, cellulose/PDMS, 2 μL Au NP–cellulose/PDMS, 20 μL Au NP–cellulose/PDMS, 200 μL Au NP–cellulose/PDMS, 20 μL Au/PDMS.

The dielectric constant of PDMS, cellulose/PDMS, 2 μL Au NP–cellulose/PDMS, 20 μL Au NP–cellulose/PDMS, 200 μL Au NP–cellulose/PDMS, 20 μL Au/PDMS, nanocomposites are shown in Fig. 7(a). The dielectric constant of pure PDMS is 1.2 at low frequencies and remains constant over the entire frequency range. The dielectric constant increases after the addition of cellulose in PDMS. The dielectric constant of cellulose/PDMS is 7.3 and 6.2 at the lowest and the highest frequency value, respectively. The rise in the dielectric constant is due to the presence of cellulose in different orientations due to which the electric field is more effective along the cellulose microfibrils throughout the PDMS matrix.^34^ Also the rise in dielectric constant in cellulose/PDMS may be attributed to the interfacial polarization at the interface between cellulose and PDMS.^35^ The 20 μL Au NP/PDMS nanocomposite sample have a low dielectric constant of 1.9. This may be attributed to the high electrical conductivity of Au NPs.^36^ It was found that the dielectric constant of 200 μL Au NP–cellulose/PDMS nanocomposite sample is 7.7 which is the highest among all the nanocomposites. The rise in the dielectric constant is attributed to the interfacial polarization at the interface between Au NPs, cellulose and PDMS. The dielectric constant is high at low frequencies due to the occurrence of interfacial and dipole polarizations occurring at low frequencies.^37^ The occurrence of interfacial polarization can be explained by Maxwell–Wagner–Sillars (MWS) effect. According to this effect a significant amount of charges accumulates at the interface of conducting filler and the insulating polymer by surface polarization, which is a consequence of different values of conductivity of the two-different materials that are present. Also, numerous micro/nano capacitors are formed in the nanocomposite due to the presence of Au NPs. It is also observed that the dielectric constant of all the Au NP–cellulose/PDMS nanocomposites falls slightly at higher frequencies. This can be attributed to the relaxation behavior of the nanocomposite at higher frequencies. This behavior is believed to be due to the interaction of the Au NPs with cellulose in the PDMS matrix.^38^ The dielectric constant of 20 μL Au NP/PDMS is slightly higher than the neat PDMS. This can be attributed to the addition of conducting Au NPs in PDMS matrix, which enhances the space charge polarization and Maxwell–Wagner–Sillars effect.^39^

In the literature it is found that the enhancement in the dielectric constant due to the addition of noble metal nanoparticles can be explained by the “boundary layer capacitor effect”. According to this effect it is assumed that a large number of equivalent elementary capacitors are formed. The conducting Au NPs behave as electrodes of the capacitor whereas the cellulose exists as an insulating dielectric layer between the electrodes. After an external electric field is applied to the system the overall dielectric constant of the nanocomposite rises.^40^

The dielectric loss of PDMS, cellulose/PDMS, 2 μL Au NP–cellulose/PDMS, 20 μL Au NP–cellulose/PDMS, 200 μL Au NP–cellulose/PDMS, 20 μL Au/PDMS nanocomposites are shown in Fig. 7(b). It is observed that the dielectric loss of pure PDMS, 20 μL Au NP/PDMS and cellulose/PDMS are 0.075, 0.04, and 0.07 respectively. This indicates that in the presence of Au NPs the dielectric loss of PDMS nanocomposite decreases. But due to the addition of cellulose in PDMS the dielectric loss does not decrease. A comparatively higher dielectric loss in PDMS and cellulose/PDMS indicates that the dipoles are more relaxed and hence move more violently under the influence of an external electric field. It is reported that dielectric loss is the proportion of conduction charge transferred to that stored by polarization. This indicates that the low dielectric loss in Au NP–cellulose/PDMS nanocomposite is suitable for piezoelectric and dielectric applications.^35^

It is observed that the dielectric loss of all the Au NP containing samples increases with frequency but later falls. This can be attributed to the fact that after a certain critical frequency (*f*_c_) the dipole oscillations becomes independent of the changing electric fields. Thus the dipole oscillations reduces with the increase in the frequency of the applied electric field above *f*_c_, simultaneously the dielectric loss also falls.^9^ The relaxation time (*τ*_rel_) was calculated by using the following relation3
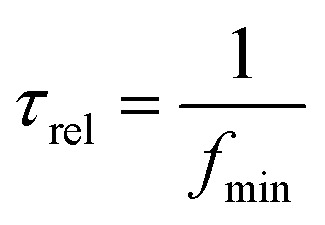
where *f*_min_ is the frequency at the lowest dielectric loss value. The relaxation times for PDMS, cellulose/PDMS and 2 μL Au NP–cellulose/PDMS were 0.92 μs. The relaxation times for 20 μL Au NP–cellulose/PDMS and 20 μL Au NP/PDMS were 0.94 μs. The relaxation times for 200 μL Au NP–cellulose/PDMS is 1 μs. This indicates that the incorporation of Au NPs makes the dipoles less relaxed. Hence, the dipoles move less violently under the influence of an external electric field in the presence of Au NPs.^41^ The dielectric loss is also dependent on the volume fraction of the conductive filler and the density of the polymer matrix. The dielectric loss of all the Au NP–cellulose/PDMS nanocomposites are less at lower frequencies due to the disruption in the ordered layered structure within the PDMS matrix. This occurs due to the introduction of defects and voids in the nanocomposite owing to the presence of Au NPs.^42^ The high dielectric constant and low dielectric loss values make Au NP–cellulose/PDMS based nanocomposites good energy storage material.

The dielectric polarization in Au NP–cellulose/PDMS arises due to the stable thermal motion of the different chemical groups present in the nanocomposite. Changes in localized charge density, isomeric transitions, rotation of side groups cannot also be ruled out in the Au NP–cellulose/PDMS nanocomposite.

**Fig. 8:**
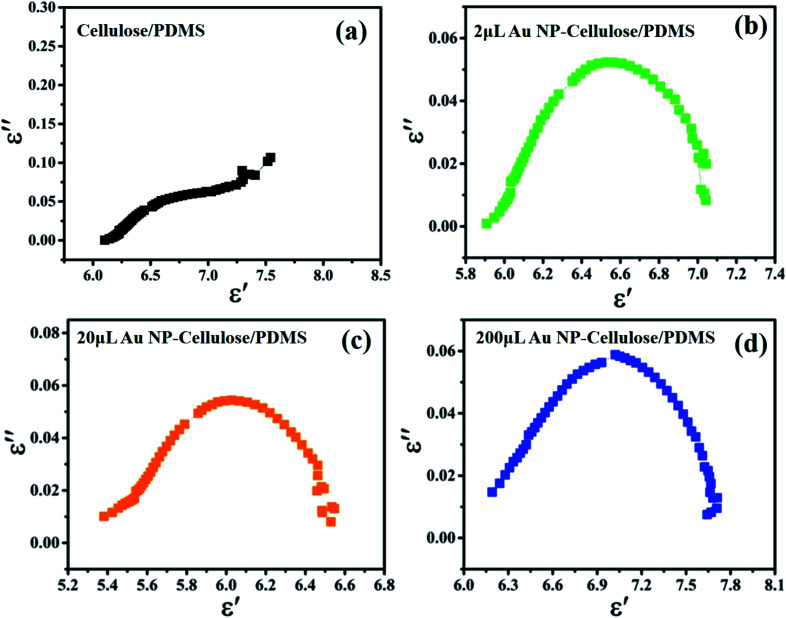
The *ε*′′ *vs. ε*′ plot of (a) cellulose/PDMS, (b) 2 μL Au NP–cellulose/PDMS, (c) 20 μL Au NP–cellulose/PDMS, (d) 200 μL Au NP–cellulose/PDMS.

The dispersed spectra in Fig. 8(a) corresponding to the *ε*′′ *vs. ε*′ plot of cellulose/PDMS indicates non-Debye relaxation process. Whereas the semicircular plot corresponding to 2 μL Au NP–cellulose/PDMS, 20 μL Au NP–cellulose/PDMS, 200 μL Au NP–cellulose/PDMS which is also known as the Cole–Cole semicircle is shown in Fig. 8(b–d), respectively. To understand the MWS effect, the study of the dipole relaxation mechanism is significant. The Debye relaxation mechanism is caused due to delay in polarization with the application of electric field within a dielectric medium. The number of Cole–Cole semi-circle in any plot is equal to the number of Debye relaxation. The equation for Cole–Cole semicircle is given as:^43^4
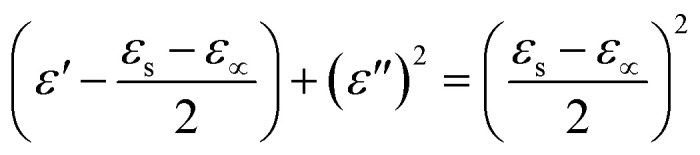


Thus, one number of Debye relaxation mechanism occur in 2 μL Au NP–cellulose/PDMS and 20 μL Au NP–cellulose/PDMS and 200 μL Au NP–cellulose/PDMS. No Debye relaxation mechanism occur in 200 μL Au NP–cellulose/PDMS and pure cellulose/PDMS. The broken Cole–Cole semicircle indicates distributed relaxation times in case of 200 μL Au NP–cellulose/PDMS and pure cellulose/PDMS.^44^

**Fig. 9:**
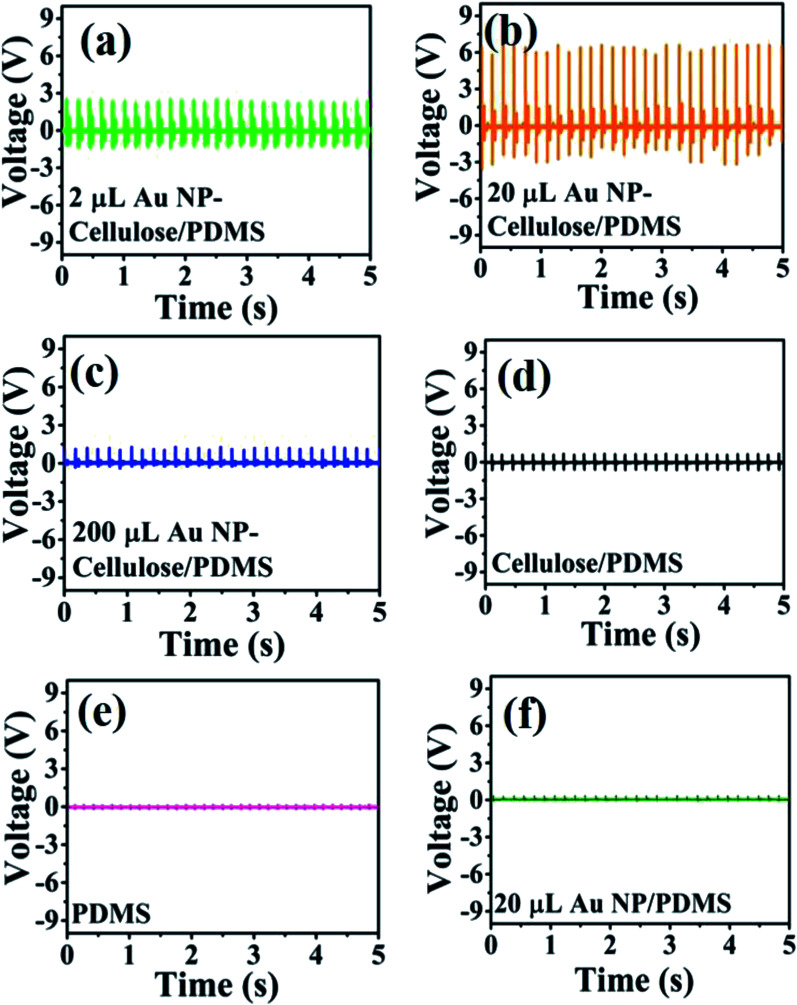
Shows the open circuit voltage of the (a) 2 μL Au NP–cellulose/PDMS, (b) 20 μL Au NP–cellulose/PDMS, (c) 200 μL Au NP–cellulose/PDMS, (d) cellulose/PDMS, (e) pure PDMS and (f) 20 μL Au/PDMS.

Mechanical energy harvesting device was fabricated from the 2 μL Au NP cellulose/PDMS, 20 μL Au NP–cellulose/PDMS, 200 μL Au NP–cellulose/PDMS, cellulose/PDMS, pure PDMS and 20 μL Au NP/PDMS nanocomposites. The mechanical energy harvesting devices were excited by a self-made mechanically operated external vibration source. The open circuit voltage outputs from all the nanocomposites are shown in Fig. 9(a–f). The open circuit voltage generated by pure PDMS is few hundred milli volts. The open circuit voltage generated by pure cellulose/PDMS composite is less than 1 V, which clearly indicates that the enhancement of the output voltage is a consequence of the interaction between Au NPs and cellulose. The 20 μL Au NP–cellulose/PDMS showed highest output voltage of nearly 6 V among all the nanocomposites. The mechanical energy harvesting performance of 2 μL Au NP–cellulose/PDMS and 200 μL Au NP–cellulose/PDMS nanocomposites were recorded and it was found that the output voltage from them is less as compared to the 20 μL Au NP–cellulose/PDMS. The less output voltage from the 2 μL Au NP–cellulose/PDMS may be attributed to the presence of lower concentration of Au NPs. Whereas the less output voltage from the 200 μL Au NP–cellulose/PDMS nanocomposites maybe either because of the agglomeration of Au NPs or due to leakage of piezoelectric charges owing to the formation of conducting pathways. The open circuit voltage recorded from the 20 μL Au NP–cellulose/PDMS was also less than 1 V, which again indicates that the enhancement in the output voltage is not only because of Au nanoparticles or due to water.

The enhancement in the open circuit voltage in the nanocomposite due to the addition of Au NPs was expected as it is reported previously that due to the nanoscale dimensions of the filler material, the interfacial area between the filler and the polymer enhances manifold. Subsequently, dipolar interface layer induced coupling effect takes place, which brings many unexpected excellent macroscopic properties in the nanocomposite like higher polarization, dielectric response, breakdown strength in some cases.^45^ However, in this case it is found that the piezoelectric effect enhances due to the possible formation of additional stress induced dipoles within the nanocomposite owing to the hydrogen bonding based interaction between the cellulose and Au NPs as indicated by the IR spectroscopy.

One of the ways to explain the enhanced output voltage is to attribute the presence of large number of additional electrons on the Au NP surface. These electrons engage with a greater number of hydrogen atoms present in the cellulose to form dipoles through electrostatic interactions. These dipoles are aligned in one particular direction when the nanocomposite is externally excited by a mechanical vibration.

The short circuit current generated from the different nanocomposites due to the excitation generated by a self-made mechanically operated vibration source are shown in Fig. 11(a–f). The voltage drops across a 10 MΩ resistance was measured by a digital storage oscilloscope and from where the current was calculated using ohm's law. 700 nA is the highest output current that is obtained from 20 μL Au NP–cellulose/PDMS. The current output from the 20 μL Au NP/PDMS is almost equal to the current output from the pure PDMS. The current output is higher in cellulose/PDMS as compared to pure PDMS which indicates the piezoelectric activity of cellulose. These results also indicate that the incorporation of Au NPs in cellulose/PDMS increases the overall current output. However, the current output from the 2 μL Au NP–cellulose/PDMS and 200 μL Au NP–cellulose/PDMS nanocomposites were less as compared to the 20 μL Au NP–cellulose/PDMS nanocomposite. The optimization of the concentration of the Au NPs in the nanocomposite is done first by using 2 μL Au NP followed by 20 μL Au NP and finally by adding 20 μL Au NP. It was found that the highest electrical output obtained is from the nanocomposite containing 20 μL Au NP.

**Fig. 10:**
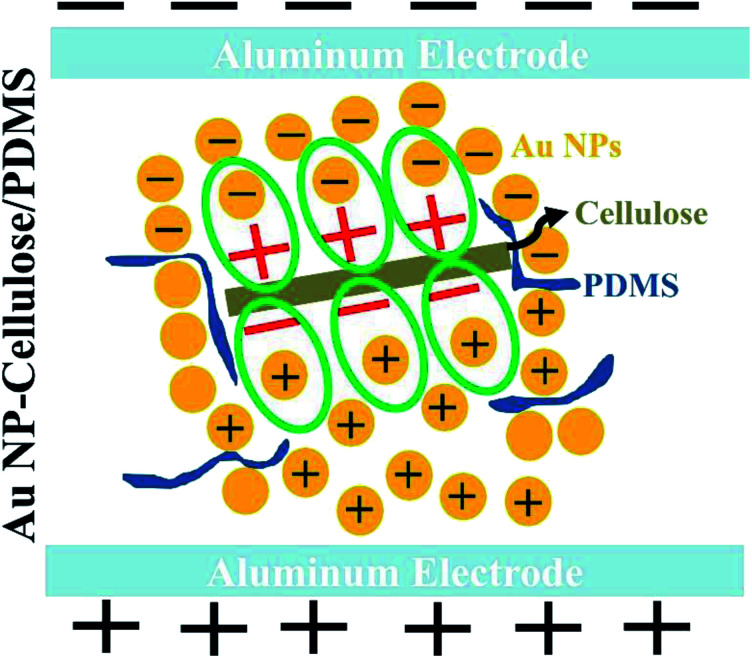
Shows the schematic indicating the formation of new dipoles between Au NP–cellulose under stress and subsequently the enhancement of the piezoelectricity of the overall nanocomposite.

The overall enhancement in the piezoelectric output voltage and current in the Au NP–cellulose/PDMS can be attributed to the model proposed by Tanaka *et al.*^46^ It includes that an enhancement in the dielectric properties is achievable with a properly engineered nanoparticle/polymer multilayered interface. The cellulose microfibrils would behave as a polar inner layer structure, that would isolate the conducting Au NPs, while the PDMS would behave as a robust, physically thick, non-polar insulating layer. Under the application of an external mechanical vibration the polar cellulose microfibrils would charge the surface of the nearby residing Au NPs and in this way contribute to a significant amount of rise in the distribution of the overall piezoelectric polarization throughout the nanocomposite. The PDMS which behaves as the non-polar insulating layer prevents the formation of any conducting paths within the nanocomposite by isolating the Au NPs which are conducting in nature. Due to this reason the dielectric constant remains high throughout and does not reduce in magnitude with the subsequent increase in the concentrations of Au NPs within the nanocomposite. Fig. 10 shows the schematic illustration of the mechanism of enhancement of the piezoelectric output when the nanocomposite is under external stress.

**Fig. 11:**
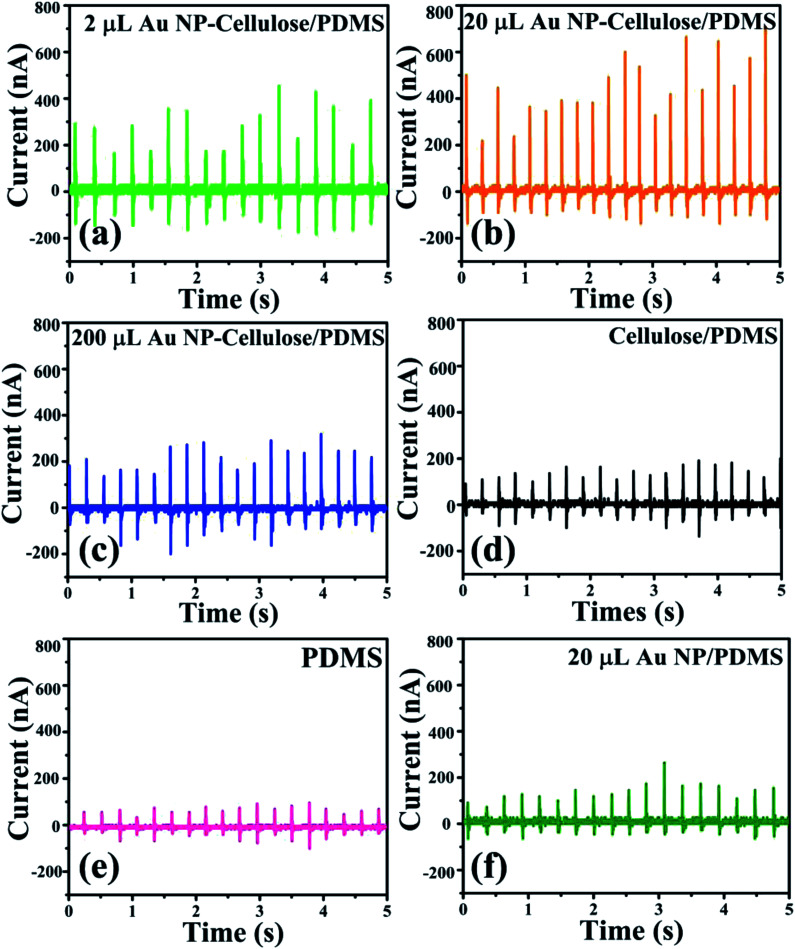
Shows the short circuit current of the (a) 2 μL Au NP–cellulose/PDMS, (b) 20 μL Au NP–cellulose/PDMS, (c) 200 μL Au NP–cellulose/PDMS, (d) cellulose/PDMS, (e) pure PDMS and (f) 20 μL Au/PDMS.

Also, due to the presence of the conducting Au NPs in the nanocomposite matrix, it is expected that the interparticle conduction mechanism would increase due to the tunneling effect. The tunneling effect arises when the percolation threshold is not attained by the conducting fillers. Percolation threshold is the condition when the conducting filler particles form a continuous path throughout the nanocomposite. The tunneling effect is dependent on the distance between the conductive fillers. The conductivity of the overall nanocomposite increases with the decrease in the distance between the Au NPs due to the tunneling effect. Due to this reason the piezoelectric voltage that is generated within the nanocomposite reaches the external electrodes efficiently without any loss.^47^ Thus the enhancement in the piezoelectric output voltage maybe attributed to the tunneling effect due to the presence of conducting Au NPs in the Au NP–cellulose/PDMS nanocomposite.^48^

To ascertain that the voltage signals from the Au NP–cellulose/PDMS nanocomposite samples are piezoelectric in nature the piezoelectric coefficient (d_33_) of the cellulose/PDMS is measured using a d_33_ meter. The d_33_ value of the cellulose/PDMS was found to be 8 pC N^−1^. To verify the correctness of the measured d_33_ value the frequency dependent capacitance of the cellulose/PDMS is measured, which is shown in Fig. S1, in the ESI.† The d_33_ value was calculated using eqn (S1), which is provided in the ESI.† The calculation indicated the d_33_ value of the cellulose/PDMS to be 8.8 pC N^−1^. This indicates that the cellulose used in this work is piezoelectric in nature and the voltage output produced from the Au NP–cellulose/PDMS nanocomposites are consistent with the piezoelectric frame of references.

It is found that the generated output voltages and currents are higher under pressing conditions and lower under releasing conditions. This might be attributed to the elasticity of the PDMS material. The elasticity forces the material to return to its original shape, irrespective of the force that is imparted.^49^

**Fig. 12:**
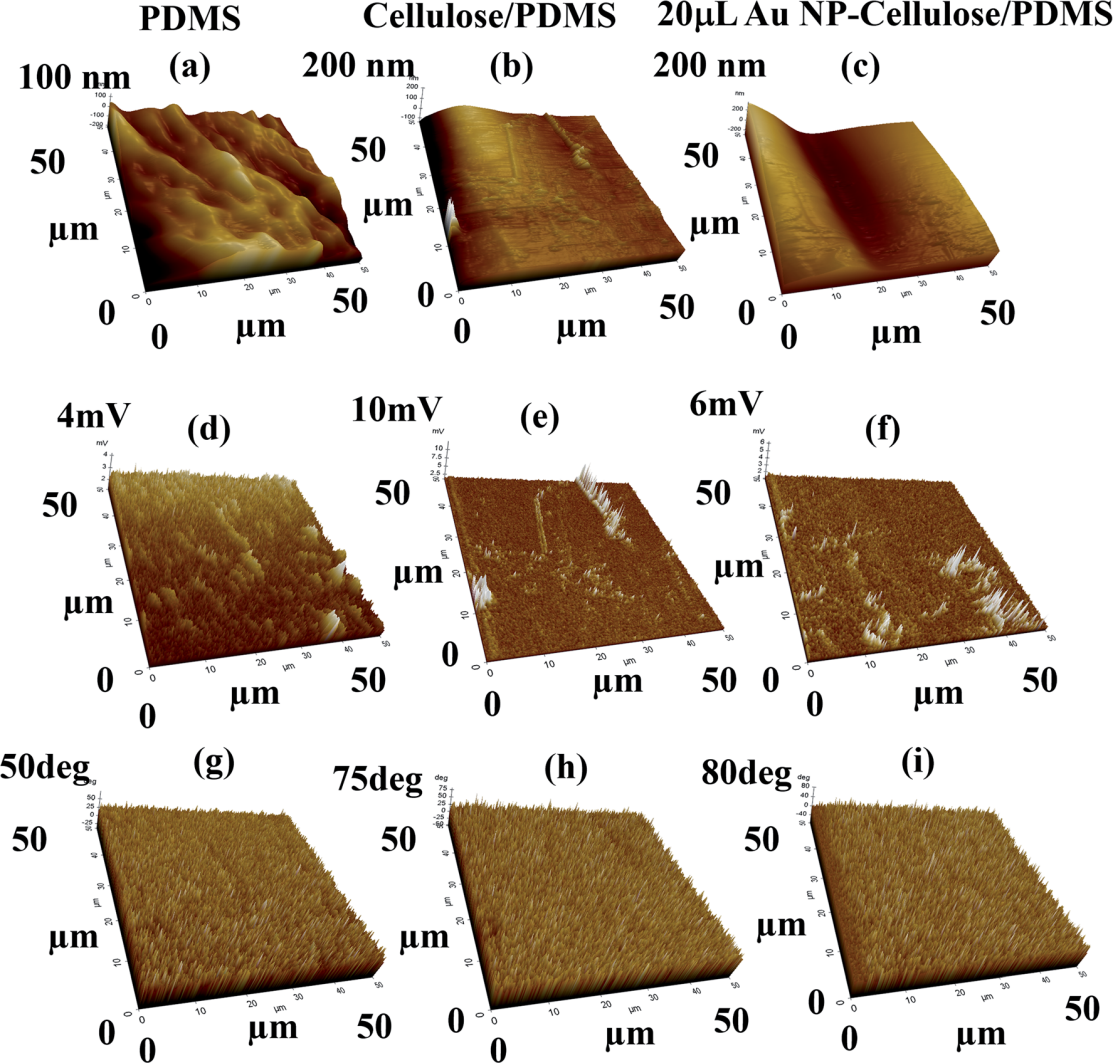
Shows the 3D PFM (a–c) height profile, (d–f) amplitude response, (g–i) phase response from the PDMS, cellulose/PDMS and 20 μL Au NP–cellulose/PDMS nanocomposite samples respectively.

Piezoresponse force microscopy (PFM) is a powerful nanoscale imaging technique which is used to explore the local piezoelectric response. The imaging mechanism of PFM is based on detecting the electromechanical response which is induced by the inverse piezoelectric effect with the help of an atomic force microscopy (AFM) cantilever tip. However, it is well known that PFM characterization is impaired by non-piezoelectric responses like electrostrictive, electrostatic effects that can be present in both contact and non-contact mode of operation.^50^ In the present study the PFM was used in the contact mode where the cantilever recorded lateral PFM signals to evaluate the electromechanical responses. To evaluate the nanoscale electromechanical response from the Au NP–cellulose based PDMS nanocomposites PFM is performed over 50 × 50 μm^2^ area. In the PFM measurement a scan rate of 1 Hz, time constant 1 ms, sensitivity 0.1 V and drive voltage of 1 V was used. Fig. 12(a–c) shows the PFM 3D height profile of the PDMS, cellulose/PDMS and 20 μL Au NP–cellulose/PDMS nanocomposites respectively. It is seen that PDMS encapsulated cellulose microfibrils are protruding out from the nanocomposite surface in Fig. 12(b and c). The root mean square (RMS) roughness of the PDMS, cellulose/PDMS and 20 μL Au NP–cellulose/PDMS nanocomposites were determined to be 38.56 nm, 17 nm and 76 nm respectively. The PFM amplitude signal is shown in Fig. 12(d−f). The voltage signal obtained from PDMS surface is electrostatic or electrostrictive in nature.^51^ This can be confirmed by the fact that PDMS is non-piezoelectric material. The electrostatic and electrostrictive effects are dependent on the properties of the material under study.^52^ The voltage signal obtained from cellulose/PDMS and 20 μL Au NP–cellulose/PDMS nanocomposite is higher than that obtained from pure PDMS. This is because the cellulose present on the surface provides direct piezoelectric response which is a first order electromechanical response in addition to the electrostatic or electrostrictive responses which are higher order response originating from the PDMS. This piezoelectric response from the cellulose that is detected here is a local effect *i.e.* it comes only from the cellulose that is in contact with the PFM cantilever tip. Also, by comparing the RMS roughness values of the nanocomposites it can be observed that the amplitude signal is independent of the roughness of the nanocomposite surface. Thus, it can be concluded that PFM does not provide a complete conclusion towards the effect of Au NPs on the piezoelectric property of cellulose within PDMS matrix in the current study. The PFM phase image which is shown in Fig. 12(g–i) indicates several orientations of the domains from which the electromechanical responses originates from the nanocomposite surfaces.^53^

**Fig. 13:**
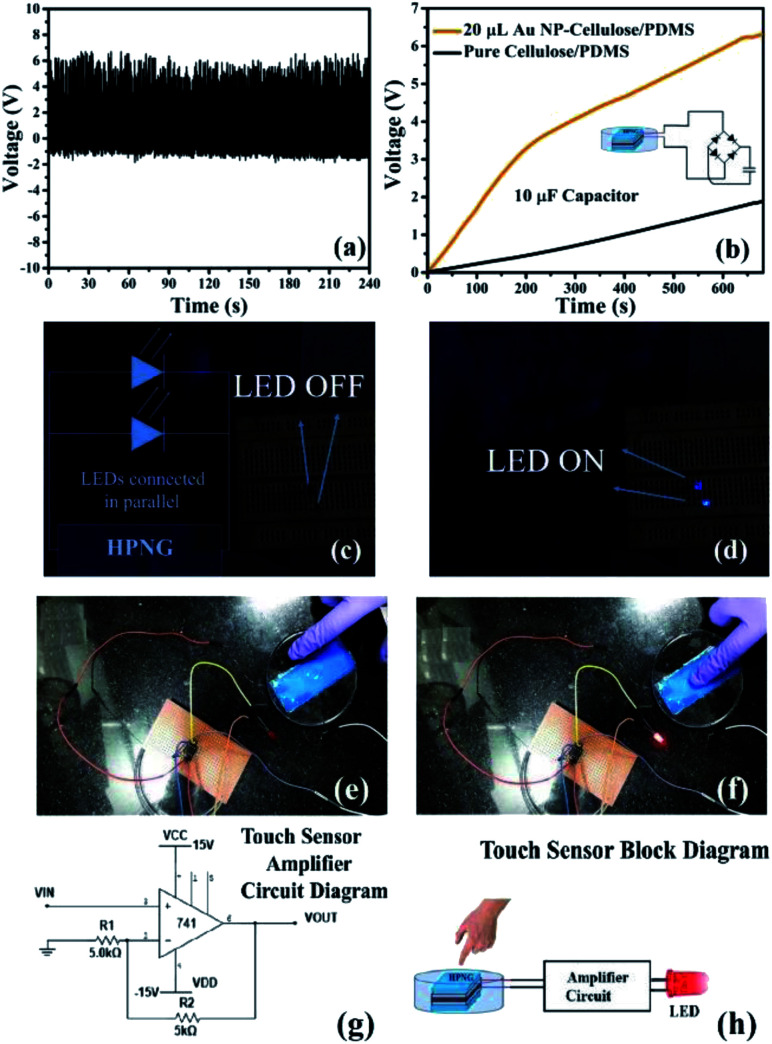
Shows the (a) stability of the PNG, (b) charging of a 10 μF capacitor by the PNG fabricated from 20 μL Au NP–cellulose/PDMS and pure cellulose/PDMS, (c) LED in off condition when PNG is not excited, (d) LED in on condition under human palm impact impartment, (e) shows the touch sensor application of the Au NP–cellulose/PDMS nanogenerator. The LED is switched off when sensor/PNG is touched in the non-sensing area, (f) the LED turns on when sensor/PNG is touched in the sensing area, (g) amplifier circuit diagram that is used to amplify the harvested current generated by the PNG, to ensure touch sensing (h) the block diagram of the touch sensor application.

The steady voltage output shows excellent stability and durability of the 20 μL Au NP–cellulose/PDMS PNG as shown in Fig. 13(a). The PNG is periodically excited and relaxed by an external electromechanical excitation source. The PNG could charge a 10 μF capacitor to 6.3 V in 677 s, whereas in the same time, the pure cellulose/PDMS based mechanical energy harvesting device could charge a 10 μF capacitor to 1 V only, which is shown in Fig. 13(b). The PNG could light blue Light Emitting Diodes (LEDs) by continuous hand striking. Fig. 13(c) shows the LED in OFF condition and Fig. 13(d) shows LED in ON condition, due to continuous human hand impulse imparting. This indicates that the PNG can be used as a probable candidate for future lighting solutions. Video 1 in the ESI† shows the LED lighting by hand impulse imparting.

Further, we demonstrated a touch sensor application of the PNG. Fig. 13(e) shows that the red LED remains turned off when the working electrode area is not touched. Fig. 13(f) shows that the red LED is turned on when the working electrode area is touched. Video 2 in the ESI† demonstrates the touch sensing application. The idea was to use the enhanced piezoelectric property of the 20 μL Au NP–cellulose/PDMS based nanocomposite to light a LED indicator every time the electrode area experiences a finger touch. The output signal generated by the PNG was amplified by an electronic amplifier circuit which is shown in Fig. 13(g). The block diagram of the touch sensor is shown in Fig. 13(h). The inputs to the amplifier are connected to the PNG, whereas the outputs of the amplifier are connected to the red LED.

To calculate the power density the PNG was excited while its two terminals were connected across a 2.2 MΩ resistance. A digital storage oscilloscope was connected across the two terminals of the resistance and a peak output voltage of 6.5 V was recorded. A peak power density of 8.34 mW m^−2^ was recorded for the PNG using the following equation:5
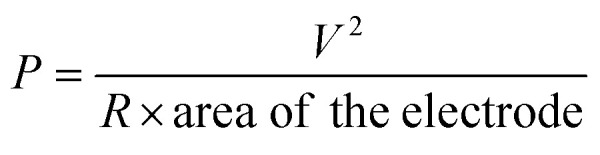
where *P* is the peak power density, *V* is the output voltage recorded, *R* is the value of the resistance.

**Fig. 14:**
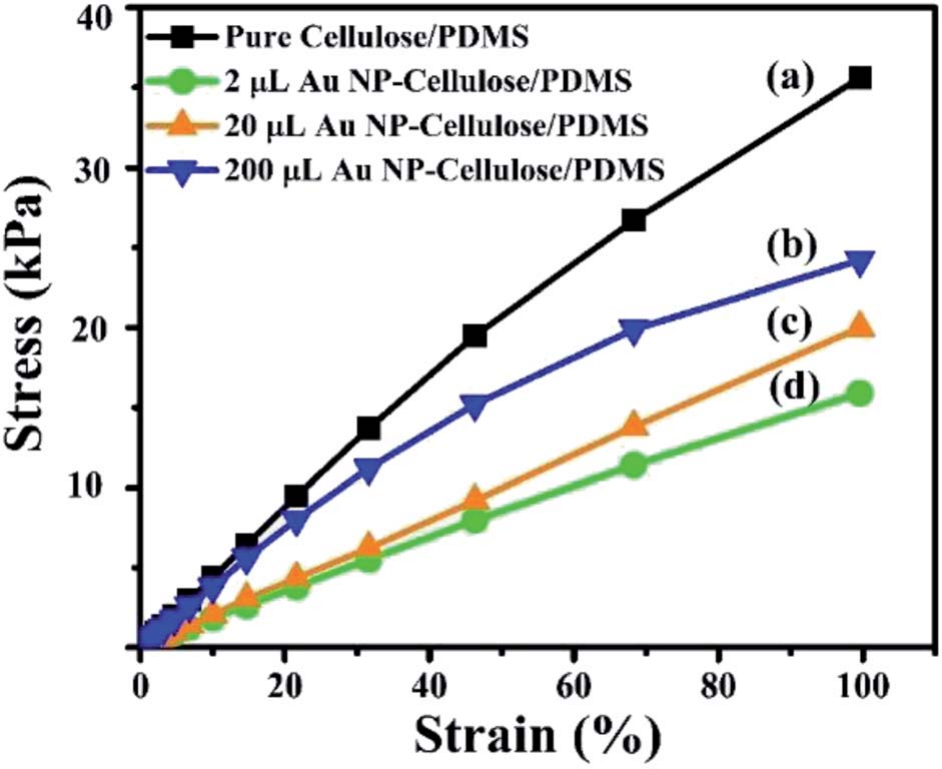
Shows the stress *vs.* strain curve of (a) pure cellulose/PDMS (b) 200 μL Au NP–cellulose/PDMS (c) 20 μL Au NP–cellulose/PDMS (d) 2 μL Au NP–cellulose/PDMS.

The stress *vs.* strain curve of all the nanocomposite samples shows non-linear behavior, as shown in Fig. 14. This is attributed to the breakage of the network fibres of cellulose, due to bond breaking or fibre pull-out. Twist in the cellulose microfibrils can also be attributed towards this behavior.^44^ It can be seen that the non-linearity follows a decreasing trend with the addition of Au NPs, this can be due to the fact that the Au NPs binds with the cellulose and prevents the damage or slippage to the cellulose microfibrils.

### Efficiency calculation

The Young's modulus of the pure cellulose/PDMS, 2 μL Au NP–cellulose/PDMS, 20 μL Au NP–cellulose/PDMS and 200 μL Au NP–cellulose/PDMS nanocomposites were found out to be 425 Pa, 326 Pa, 203 Pa, 173 Pa, respectively from the stress *vs.* strain curve.

The energy harvesting efficiency of the PNG during capacitor charging is calculated next. The input energy (*W*_in_) provided to the PNG while charging the capacitor during one cycle is given by6
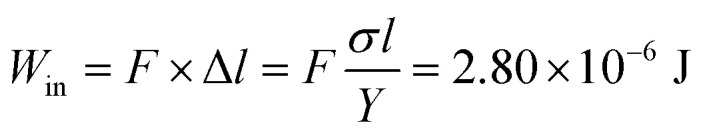
where *F* is the applied force on the PNG, which is measured externally to be 3N. Δ*l* is the deformation of the PNG when stress *σ* is 9.4 kPa and Young's modulus, *Y* is 20.2 kPa and the average thickness of the nanocomposite film *l* is 2 mm.

The total input mechanical energy transfer to the PNG during the capacitor charging is given by7
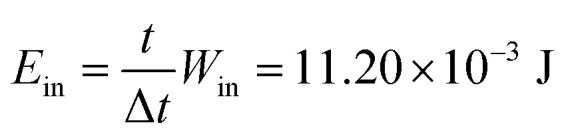
where *t* is the total time required to charge the capacitor, which is 677 s. Δ*t* is the average time duration between the two consecutive voltage peaks of the imparted pressure cycles by the mechanical excitation generation source, which is 0.172 s.

The electrical energy stored in the capacitor is given by the following equation.8
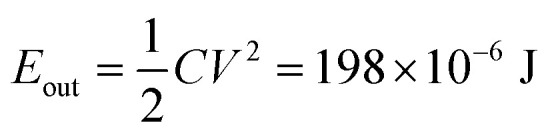
where *C* is the capacitance of the capacitor, which is 10 μF, *V* is the total voltage stored by the capacitor, which is 6.3 V.

The overall energy efficiency (*η*) is found out from the ratio of the electrical energy stored 
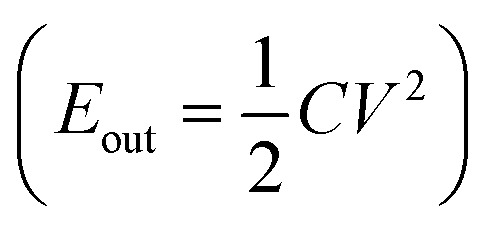
 in the 10 μF capacitor while charging and the total mechanical energy used (*E*_in_) to charge the 10 μF capacitor.9
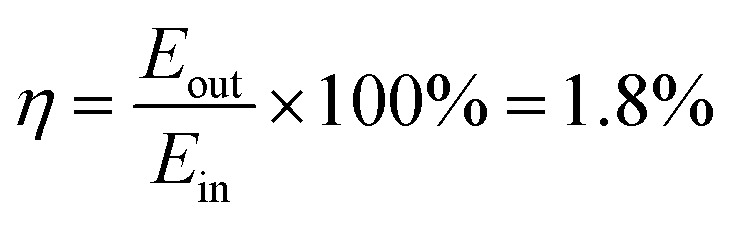


The 1.8% energy conversion efficiency obtained in this work is quite higher than some of the similar recent reported works. Table 2 shows the comparison of the efficiency of the recently reported works. Also, Table 2 indicates the fact that the poling process is followed in the process of fabrication of different piezoelectric mechanical energy harvesting devices. In contrast to the previously reported works the PNG discussed in this work delivers superior mechanical energy harvesting capability, without following any external poling steps.

**Table 2:** Performance details of previously reported data on energy harvesting devices

Sl. No.	Name of the piezoelectric device	Poling voltage and duration	Input source	Voltage (V)	Energy conversion efficiency (%)
1	Cellulose–ZnO^54^	Data unavailable	Ultrasonic bath	80 mV	—
2	ZnO NWs/PVDF^55^	100 kV mm^−1^	Linear motor	0.2 V	—
3	BaTiO_3_ NPs^56^	100 kV cm^−1^; 20 h	Bending stage	3.2V	—
4	BaTiO_3_ nanotubes^57^	80 kV cm^−1^; 12 h	Linear motor	5.5 V	—
5	PVDF-TrFE film/graphene oxide^58^	30 mV m^−1^, 1 h	Hydraulic/mechanical fatigue tester	4.3 V	—
6	Fe–RGO/PVDF^47^	Not poled	Human hand punching	5.1 V	—
7	Fe–RGO/PVDF^9^	Not poled	Human finger tapping	1.2 V	—
8	CNT/PVDF^9^	Not poled	Human finger tapping	2.5 V	—
9	PVDF/cellulose^59^	Not poled	Data not available	6.3 V	—
10	ZnO/cellulose^60^	Not poled	Smart shaker	908 mV	—
11	rGO–Ag/PVDF^27^	Not poled	Human hand tapping	18V	0.65
12	ZnSnO_3_ (ref. 61)	Not found	Human finger press	40 V	1.17
13	KNbO_3_ nanowires^62^	5.0 kV mm^−1^; 1 h	Bending tester	10.5 V	0.9
**14**	**Au NP–cellulose/PDMS**	**Not poled**	**Mechanical excitation source**	**6 V**	**1.8**

Although different device structures were previously reported for mechanical energy harvesting applications like cantilever type arrangement,^63^ cymbal structure,^64^ stack structure,^65^ micro-nano structures,^66^ however in this work we have followed the electrode-composite-electrode stack structure. Previously, Karan *et al.* demonstrated good piezoelectric energy harvesting using this type of structure.^47,49^ Cantilever type arrangement is not suitable for flexible piezoelectric devices and also the span length cannot be long. The maximum force that can be applied is also less and hence the output voltage will also be less. The disadvantage with cymbal type of systems are that it has low sensitivity to ambient sources. Also, cymbal systems have a tendency to get deformed under repeated stress.^67^ The disadvantage of micro-nano structured piezoelectric materials are that they require excitation sources like AFM, which is not feasible for practical application purposes.^1^

**Fig. 15:**
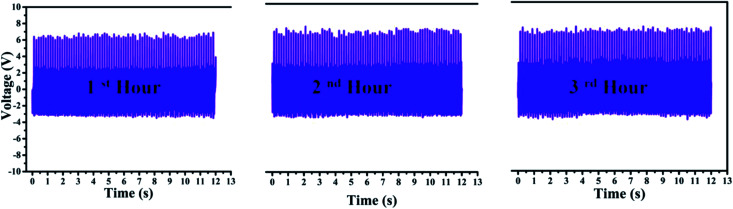
Shows the stability of the PNG when tested with a mechanically operated piezoelectric excitation source consecutively for three hours.

To examine the long-term usage, the PNG was mechanically excited by an external excitation source for three consecutive hours, as shown in Fig. 15. For the first, second and the third hour no change in the magnitude of the output voltage is observed. This indicates the stability and good lasting capability of the PNG under repeated stress for prolonged period and highlights the advantage of using the PNG for body/apparel wearable applications for enhanced period. The good durability of the PNG is achieved due to the PDMS encapsulation.

## Conclusions

In summary we have demonstrated the synthesis of Au NP–cellulose/PDMS based flexible nanocomposite with a good dielectric constant and low dielectric loss. The incorporation of Au NPs with ∼15 nm diameter in PDMS along with cellulose is found to have shown promising capability towards realizing efficient energy conversion and storage applications. It is found that cellulose/PDMS is a good dielectric material. Further with the inclusion of Au NPs in the cellulose/PDMS nanocomposite the dielectric loss reduces further. More importantly it was found that the piezoelectric output voltage and the current enhances due to the incorporation of Au NPs in the nanocomposite. The enhancement in the piezoelectricity is attributed to the overall enhancement in the dielectric property of the nanocomposite. A mechanical energy harvesting device (PNG) is fabricated with the Au NP–cellulose/PDMS nanocomposite. The PNG showed an open circuit voltage of ∼6 V when excited with a periodic force of 3 N and a short circuit current of ∼700 nA. The PNG could charge a 10 μF capacitor to 6.3 V in 557 s and showed an energy conversion efficiency of 1.8%. The PNG could light two commercial blue LEDs by direct human hand impulse impartment. A touch sensor application with the PNG is also shown. The advantage of cellulose based nanogenerator is that it can open up the possibility towards the fabrication of lead free piezoelectric nanogenerator. As cellulose is biocompatible, biodegradable and recyclable it can open the possibility to develop body implantable energy harvesting devices that can be used to harvest mechanical energy from human body movements, blood circulations *etc*.

## Conflicts of interest

The authors declare no competing financial interests.

## Supplementary Material

RA-010-C9RA10811D-s001

RA-010-C9RA10811D-s002

RA-010-C9RA10811D-s003
