# Supplementary material for: Gold nanoparticle–cellulose/PDMS nanocomposite: a flexible dielectric material for harvesting mechanical energy
Source: RSC Adv. 2020 Mar 10;10(17):10097–112. doi: 10.1039/c9ra10811d (PMC9050361; doi:10.1039/c9ra10811d)
Supplement: RA-010-C9RA10811D-s001 [file RA-010-C9RA10811D-s001.pdf]

Electronic Supplementary Material (ESI) for RSC Advances.
This journal is © The Royal Society of Chemistry 2020

Gold Nanoparticle-Cellulose/PDMS Nanocomposite: A Flexible Dielectric Material for Harvesting Mechanical Energy

Manojit Pusty and Parasharam M. Shirage *

Discipline of Metallurgy Engineering and Materials Science, Indian Institute of Technology Indore, Simrol, Indore 453552. India

* Corresponding Author E-mail: paras.shirage@gmail.com, pmshirage@iiti.ac.in.
Tel.: **+91-787-5222-331**

Piezoelectric coefficient measurement:

The piezoelectric coefficient (d_{33}) value of the Cellulose/PDMS was measured to be 8 pC/N. The d_{33} coefficient was measured using a SIN0CERA YE2730A d_{33} Meter under the condition of constant applied force of 0.5 N and frequency at 50 Hz.

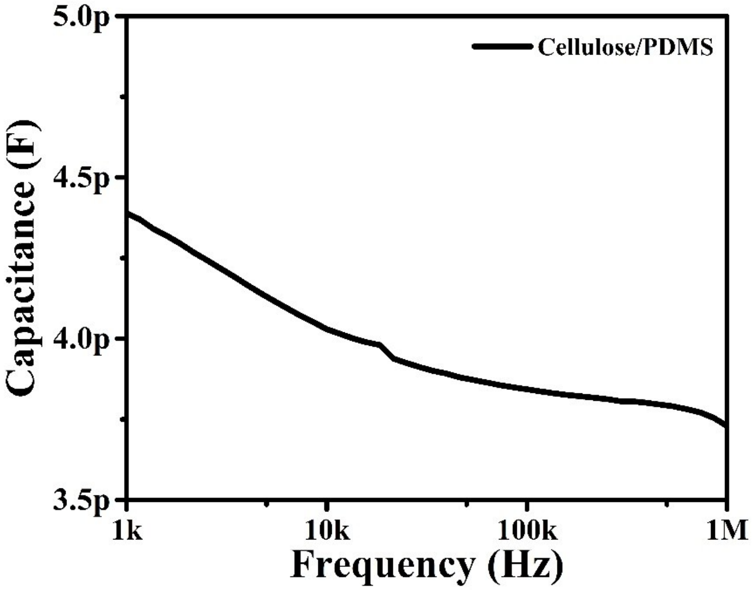

Figure S1. The variation of capacitance versus frequency curve of the Cellulose/PDMS composite used for HPNG fabrication.

The d_{33} value of Cellulose/PDMS was calculated from the capacitance vs frequency characteristics, which is shown in Figure S1. The capacitance vs frequency characteristics was measured using a NF ZM2376 LCR Meter.

To verify the measured d_{33} value a frequency dependent capacitance measurement was carried out using a NF ZM2376 LCR Meter. The following equation was used to calculate the d_{33} values:

$$d_{33} = \frac{V \times C}{F} \qquad \text{---------} \mathbf{S1}$$

Where, V is the output voltage from the Cellulose/PDMS which is found to be 6V, C is the capacitance of the Cellulose/PDMS at 1000 Hz frequency which is found to be 4.4 pF and F is the Force (3N) applied on the nanocomposite. The d_{33} coefficient of the Cellulose/PDMS was found to be 8.8 pC/N.
